# Chlorhexidine – a commonly used but often neglected culprit of dialysis associated anaphylactic reactions (case report)

**DOI:** 10.1186/s12882-021-02646-x

**Published:** 2022-01-06

**Authors:** Jia Neng TAN, Yi DA, Sabrina HAROON, Titus LAU

**Affiliations:** Division of Nephrology, University Medicine Cluster, National University Health System Republic of Singapore, Level 10, NUHS Tower Block, 1E Kent Ridge Road, Singapore, 119228 Republic of Singapore

**Keywords:** Chlorhexidine allergy, Dialysis, Dialyser reactions, case report

## Abstract

**Background:**

Hemodialysis-associated anaphylactic reactions are rare and frequently complex in nature due to the sheer number of possible culprit agents. Unfortunately, dialysis is often unavoidable or strictly essential for life-saving solute clearance or fluid removal in patients with end stage kidney failure and those with severe acute kidney injury. It is of utmost importance that the culprit agent is identified and avoided to allow continuation of dialysis treatment as needed.

**Case presentation:**

We present 2 cases of hemodialysis-associated anaphylactic reactions. These patients developed anaphylactic reactions peri-dialysis and were initially suspected to have dialyser reactions. They were investigated in a controlled healthcare setting and possible culprit agents were systemically identified and eliminated. They both underwent allergy testing and were diagnosed with chlorhexidine allergy. Of note, Case 1 was an incident dialysis patient at the time of presentation and Case 2 was a prevalent dialysis patient. This suggests that the time from initial sensitization to reaction may not always be helpful in determining if a particular agent is the culprit of an anaphylactic reaction.

In both cases, the patients were dialysed through a tunnelled dialysis catheter. We postulate that the presence of an exit site, which represents a compromise to the integrity of the skin’s epidermal barrier, may have a significant role in the development of these reactions.

As chlorhexidine is a widely used disinfectant in hemodialysis, it is imperative that we consider it as a possible culprit agent when these reactions arise. To our knowledge, there are no other reported cases of anaphylaxis secondary to chlorhexidine use in dialysis patients other than a previous report in 2017.

Our report also highlights the possibility of these reactions occurring more frequently in patients with damaged epidermal barriers and in patients exposed to higher environmental concentrations of chlorhexidine. These are novel concepts that can be explored with further research.

**Conclusion:**

Chlorhexidine associated anaphylactic reactions can occur in the peri-dialysis setting and a high index of suspicion is paramount to diagnosis.

**Supplementary Information:**

The online version contains supplementary material available at 10.1186/s12882-021-02646-x.

## Background

Hemodialysis-associated anaphylactic reactions are rare and are often complex [1] in nature due to the sheer number of possible culprit agents. In some cases, the culprit agents remain unidentified [2]. Unfortunately, patients who suffer from these reactions still require dialysis for solute clearance or fluid removal. Hence, it is paramount that the culprit agent is identified early so that the patient will be able to receive critical and adequate dialysis.

Dialysis-associated allergic reactions are generally uncommon [3]. Two main types of reactions occur – Type A and Type B reactions. Type A reactions are IgE-mediated and generally occur within the first few minutes of dialysis. They can result in various symptoms that range from mild to severe, including anaphylactic type reactions that can be life-threatening. Type B reactions are complement-mediated and generally begin in the first 15 to 30 min of dialysis. Anaphylaxis in type B reactions is rare [4]. Previously reported common inciting agents include ethylene oxide, dialyser membranes, erythropoietin stimulating agents, intravenous iron, and heparin [3].

Chlorhexidine is widely used as a skin disinfectant. It can also be found in personal and dental hygiene products. First discovered in the 1950s, it has been increasingly used as a disinfectant as it is highly efficacious against bacteria, fungi, and viruses [5]. Over the years, chlorhexidine related anaphylaxis is becoming more recognized in the setting of perioperative care [6–8], and following the use of chlorhexidine coated central venous catheters [9]. In the field of hemodialysis, life-threatening anaphylactic shock due to chlorhexidine is rare even though chlorhexidine intolerance related to contact dermatitis is common [10]. Here, we present 2 cases of anaphylactic reactions due to chlorhexidine exposure peri-hemodialysis. One of the cases occurred in an incident dialysis patient and the other case occurred in a prevalent dialysis patient. To our knowledge, other than a previous case report by Bahal et al. in 2017 [11], there are no other reported cases of anaphylaxis secondary to chlorhexidine use in a hemodialysis population.

## Case 1 (incident Dialysis patient)

Case 1 was a 65-year-old Chinese lady with a background history of advanced chronic kidney disease. She was admitted for acute on chronic kidney injury requiring dialysis. During her admission, she was not started on new medications. She had a known drug allergy towards Penicillins but does not recall the details of the allergic reaction. Her chronic medications include insulin, frusemide, calcium acetate, atorvastatin, renal vitamin, glipizide, bisoprolol, omeprazole, alfacalcidol, cholecalciferol, gabapentin and intravenous erythropoeitin. She underwent her first hemodialysis session via a non-chlorhexidine coated dialysis catheter in view of uremia and metabolic acidosis. She was dialysed for 2.5 h, at a blood flow rate of 150 ml/min and dialysate flow rate of 300 ml/min. A Polysulfone dialyser (Fresenius Medical Care, F6HPS) was used. Dialysis was anticoagulation free in view of recent catheter insertion and the session was event-free.

At her second dialysis treatment, the patient was given a 500-unit Heparin bolus, followed by a 500-unit/hour maintenance dose of Heparin to improve circuit patency. However, she developed generalized pruritus after an hour and Heparin was stopped. She completed 3 h of dialysis. Similarly, a Polysulfone dialyser was used and blood flow and dialysate flow rates were maintained at 150 ml/min and 300 ml/min respectively.

She was then given a 2-day break before her next dialysis session. Unfortunately, she developed shortness of breath, flushing, and tachycardia 2 min into her 3rd dialysis session. The treatment had to be terminated. It was anti-coagulation free as there were suspicions that Heparin might have prompted the development of pruritis in the last treatment. She was given intravenous (IV) hydrocortisone and intravenous diphenhydramine emergently with complete resolution of symptoms.

Concerns of a dialyser reaction arose and hence, she was next dialysed with a cellulose triacetate dialyser (Nipro Sureflux 19E). Heparin was avoided and the dialysis catheter was locked with citrate instead. The session was uneventful. Table 1 summarises her subsequent dialysis sessions. She continued to receive her usual medications during her inpatient stay and a review of her drug chart was performed to identify potential culprits. She was not taking ACE-inhibitors and was not on any new medications. Full blood counts performed did not reveal the presence of eosinophilia.

**Table 1:** Details of renal replacement therapy conducted during the patient’s hospital stay. Includes cleansing solution, anticoagulation agent and catheter locking solutions used

Session	Location	Modality	Cleansing Solution	Dialyser	Anticoagulation	Catheter Locking Agent	Reaction
1	Inpatient Dialysis Center	LED (Low Efficiency Dialysis)	Chlorhexidine	FMC, F6HPS	None	Heparin	Nil
2	Inpatient Dialysis Center	LED	Chlorhexidine	FMC, F6HPS	Heparin	Heparin	Pruritis
3	Inpatient Dialysis Center	LED	Chlorhexidine	FMC, F6HPS	None	Citrate	Shortness of breath and flushing after 2 min. Dialysis terminated. IV Hydrocortisone and IV Diphenhydramine given
4	Inpatient Dialysis Center	LED	Chlorhexidine	Nipro Sureflux 19 E	None	Citrate	Nil
5	Inpatient Dialysis Center	HD	Chlorhexidine	Nipro Sureflux 19 E	Heparin	Citrate	Nil
6	Inpatient Dialysis Center	IsoUF	Chlorhexidine	Nipro Sureflux 19 E	None	Citrate	Nil
7	Inpatient Dialysis Center	HD	Chlorhexidine	Nipro Sureflux 19 E	None	Heparin	Nil
8	Inpatient Dialysis Center	HD	Chlorhexidine	Nipro Sureflux 19 E	Heparin	Heparin	Nil
9	Inpatient Dialysis Center	IsoUF	Chlorhexidine	Nipro Sureflux 19 E	Heparin	Heparin	7 min into dialysis – generalised erythema / pruritis / angioedema. No stridor / wheeze. Blood pressure remained within normal range. Dialysis was terminated. IV Hydrocortisone and IV Diphenhydramine given

Abbreviations: *LED* Low Efficiency Dialysis

She was dialysed successfully for the next 5 sessions without issues but developed generalized erythema, pruritis and angioedema 7 min into her 9th dialysis session.

In view of the previous reactions during dialysis and unclear etiology of anaphylaxis on dialysis, the decision was made to transfer her to a tertiary hospital with an established allergy service. Serum tryptase level was sent and was not elevated. This was likely due to a significant delay of more than 48 h between the occurrence of the reaction and sampling of serum tryptase level.

She was reviewed by an allergist after her transfer and dialysis was held off temporarily for investigation. She was managed with high doses of diuretics. A review of her previous dialysis sessions did not reveal any clear inciting agents of anaphylaxis. The Polysulfone dialyser (FMC, F6HPS) used was steam sterilized, and Nipro Sureflux 19E was sterilized with gamma radiation. None of the dialysers underwent ethylene oxide sterilization. She was planned for a skin prick test and intradermal test after anti-histaminergic effects from prescribed anti-histamines had worn off. As the inciting cause of her previous reactions was not clear, care was made to avoid latex gloves, chlorhexidine and heparin at her next dialysis session. She was dialysed for 2 h with a Nipro Sureflux 19E dialyser and the session was, fortunately, uneventful.

She subsequently underwent allergy testing. Her intradermal test returned positive for chlorhexidine. In view of the findings, she was gradually restarted on heparinized dialysis, and a Polysulfone dialyser was reintroduced. She is now dialyzing without further issues at an outpatient center.

## Case 2 (prevalent Dialysis patient)

Case 2 is a 60-year-old lady with diabetic nephropathy who was started on hemodialysis 8 months ago. She was electively admitted for transposition of her brachiobasilic arteriovenous fistula under general anesthesia. The patient is known to develop rash and pruritis with nifedipine use and was not started on new medications during her admission. Her chronic medications include aspirin, atorvastatin, calcium acetate, frusemide,

bisoprolol, omeprazole, glipizide, renal vitamin and intravenous erythropoietin. Postoperatively she underwent routine dialysis in the hospital’s Inpatient Dialysis Center (IDC). However, she turned unwell and developed severe hypotension 2 min into dialysis. Treatment was terminated immediately, and she was transferred to the Intensive Care Unit (ICU) for Continuous Renal Replacement Therapy (CRRT).

On arrival to the ICU, her clinical condition improved spontaneously with no further need for ventilatory or circulatory support. Intermittent hemodialysis was attempted the next day in ICU and it was uneventful. She was then transferred back to the general ward. Two days later, dialysis was reattempted at IDC. Once again, she developed severe hypotension, hypoxia, and an urticarial rash over her arms and neck. Dialysis was terminated and she was sent back to the ICU. Again, there was an immediate improvement in her clinical status upon arrival to ICU, and her subsequent dialysis session in ICU the day after was unremarkable. She was then transferred back to the general ward. Serum Tryptase sent within 4 h of the reaction was elevated, suggesting a hypersensitivity reaction. However, the list of possible culprit agents was extensive, and the one responsible remained unidentified.

Unfortunately, during her next dialysis session at IDC, she developed hypotension, hypoxia, and generalized urticaria before the dialysis nurses connected her catheter to the dialysis machine. She was emergently treated with Intramuscular (IM) adrenaline, IV hydrocortisone, IV diphenhydramine and saline infusion. This allowed us to narrow down the list of differentials to skin disinfectants, possible environmental triggers, and dialysis catheter locking solutions.

A skin prick test was performed given the above, and it indicated sensitization to Chlorhexidine at various concentrations and dilutions. Other agents that tested negative include citrate, environmental cleansing agents and common aeroallergens (dust and fungal). Subsequent dialysis in a single room in the general ward and then in IDC with Povidone Iodine as disinfectant remained uneventful.

Details of each session are listed in Table 2 as follows:

**Table 2:** Details of renal replacement therapy conducted during the patient’s hospital stay. Includes renal replacement therapy modality, cleansing solution, anticoagulation agent and catheter locking solutions used

Session	Location	Modality	Cleansing solution	Dialyser	Anticoagulation	Catheter locking solution	Change of exit site dressing performed	Reaction
1	Inpatient Dialysis Center	HD	Chlorhexidine	FMC F7 HPS	None	Citrate	Yes, to Biopatch®	Hypotension. IV Saline given
2	Intensive Care Unit	CRRT	Chlorhexidine	Gambro Prismaflex	Heparin	Citrate	No	Nil
3	Intensive Care Unit	HD	Chlorhexidine	FMC F7 HPS	Heparin	Citrate	No	Nil
4	Inpatient Dialysis Center	HD	Chlorhexidine	FMC F7 HPS	Heparin	Citrate	No	Hypotension, Hypoxia, Urticaria. IV Saline given. IV Dopamine infusion started.
5	Intensive Care Unit	HD	Chlorhexidine	FMC F7 HPS	Heparin	Citrate	No	Nil
6	Inpatient Dialysis Center	HD	Chlorhexidine	FMC F7 HPS	None	Citrate	Yes, to Biopatch®	Hypotension, Hypoxia, Urticaria before dialysis catheter was connected to machine. IM Adrenaline, IV Diphenhydramine, IV Hydrocortisone, IV Saline given
7	Intensive Care Unit	IsoUF+HD	Chlorhexidine	FMC F7 HPS	Heparin	Citrate	No	Nil
8	Intensive Care Unit	IsoUF+HD	Chlorhexidine	FMC F7 HPS	Heparin	Citrate	Yes	Nil
9	General Ward (Single room)	HD	Povidone Iodine	FMC F7 HPS	Heparin	Citrate	Yes	Nil
10	General Ward (Single room)	HD	Povidone Iodine	FMC F7 HPS	Heparin	Citrate	Yes	Nil
11	Inpatient Dialysis Center	HD	Povidone Iodine	FMC F7 HPS	Heparin	Citrate	Yes	Nil
12	Inpatient Dialysis Centre	HD	Povidone Iodine	FMC F7 HPS	Heparin	Citrate	Yes	Nil

Abbreviations: *HD* Hemodialysis, *CRRT* Continuous renal replacement therapy, *IsoUF* Isolated ultrafiltration. *Biopatch®*: a polyurethane foam disc impregnated with chlorhexidine gluconate

On further review of the patient’s clinical presentation and history, it is interesting to note that her outpatient dialysis center had initially used Povidone Iodine as a cleansing solution. Three months before her admission, they had switched their cleansing agent to Chlorhexidine. Following this, there were occasional intradialytic hypotensive episodes that resolved spontaneously and she was able to complete her treatment sessions without the need for further escalation of care or hospitalization. She was discharged with strict instructions to avoid Chlorhexidine for all dialysis treatments and has since been doing well.

## Discussion and conclusions

Chlorhexidine allergy is a rare cause of dialysis-related hypersensitivity reactions, but its effects can be devastating, especially in Type A reactions shown in the case scenarios presented above. The patient in Case 1 was able to tolerate some sessions of dialysis with exposure to chlorhexidine with no associated reaction. Such a phenomenon was observed in a previous case report [1]. It was then postulated that patients who have experienced a reaction to an allergen might be able to tolerate re-exposure shortly after the acute event due to the refractoriness of the system. It is also possible that she was exposed to lower amounts of chlorhexidine in those sessions that she tolerated.

Based on the above 2 scenarios presented, it seems that the onset of an anaphylactic reaction from the time of initial sensitization of Chlorhexidine varies. In Case 2, the patient had a sensitization period of 3 months whereas the reaction occurred within days in Case 1. This suggests that a longer length of time from initial sensitization does not exclude an agent’s possibility of being the culprit of an anaphylactic reaction.

In both cases, the patients were dialyzed through a tunnelled dialysis catheter. The presence of an exit site, which represents damage to the skin’s epidermal barrier increases exposure to culprit agents and contributes to the development of these reactions. A review by Heinemann et al. [12] highlighted the possibility of heightened immediate hypersensitivity reactions that occur when chlorhexidine is applied to damaged epidermal barriers or mucosal membranes.

Allergen concentration appears to be an important factor in Case 2. Despite the use of chlorhexidine, the patient tolerated the dialysis sessions in ICU (Sessions 2,3,5,7,8) well. However, when she was dialysed in IDC & chlorhexidine was used, she would inadvertently develop an anaphylactic reaction. The most striking difference would be the distance between dialysis machines - dialysis machines in IDC are placed approximately 2 m apart with no physical barrier separating stations. In the ICU setting however, the patient was dialysed in a single room. There is likely increased aerosolized chlorhexidine concentrations in IDC (open setting, multiple patients) as compared to the ICU. The association between increased aerosolised concentrations of chemical compounds and sensitization was previously shown in a study by Choi et al. [13]

We believe that the chlorhexidine-impregnated Biopatch® placed over the patient’s (Case 2) catheter exit site also contributed to the reactions. The increased focal concentration at the exit site might have further contributed to her overall chlorhexidine exposure, increasing her chance of developing an anaphylactic reaction. This could possibly explain why she tolerated dialysis at her satellite center but not during her inpatient stay. As both patients had end stage kidney disease, they did not have significant residual renal function and did not have urinary catheters placed.

Serum Tryptase levels were sent off in both cases and returned positive only in Case 2. The negative result elicited from Case 1 was likely due to a significant delay between the reaction and sampling of serum for Tryptase serology. Sampling of specific IgE antibodies towards Chlorhexidine or drug basophil activation tests would have been helpful in establishing the diagnosis [14]. Unfortunately, this is limited by availability of the test in our unit and samples were not taken from both patients in our cases.

**Fig. 1:**
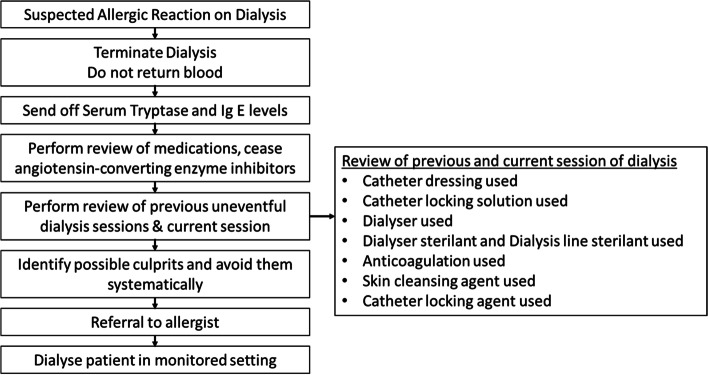
Flowchart for the systematic evaluation of dialysis-related allergic reactions

We anticipate that chlorhexidine-induced reactions will increase following the switch to chlorhexidine-based agents as first line agents in dialysis units. This is likely as there is evidence of superiority of chlorhexidine-based products in reduction of catheter-related blood stream infections [15, 16]. These reactions may initially appear to be “dialyser related” reactions. The management of patients with suspected dialyser reactions should prompt a review of hemodialysis related materials use and a careful evaluation of exposure to sterilization/cleansing agents. A high index of suspicion is required for prompt and accurate identification of the inciting agent. We have attached below a flowchart for the systematic evaluation of dialysis-related allergic reactions (Fig. 1). In patients with established allergy towards chlorhexidine, povidone iodine is used as second line agent [17].

Future research in this area could potentially focus on delineating the incidence of such reactions in patients dialyzing with catheters compared to those dialyzing via arteriovenous fistulas/grafts. This would enable us to evaluate the association between damaged epithelial membranes and allergic reactions in greater detail.

Subsequent studies may also consider evaluating aerosolized concentrations of Chlorhexidine in different environments and its consequent effects on allergic reactions.

## Supplementary Information

**Additional file 1.** CARE Checklist of information to include when writing a case report.

## Data Availability

Not applicable.

## Abbreviations

HD: Hemodialysis
CRRT: Continuous renal replacement therapy
IsoUF: Isolated ultrafiltration
LED: Low efficiency dialysis.
Biopatch®: A polyurethane foam disc impregnated with chlorhexidine gluconate
IDC: Inpatient Dialysis Center
ICU: Intensive Care Unit
