# Correction to: Chlorhexidine – a commonly used but often neglected culprit of dialysis associated anaphylactic reactions (case report)

**DOI:** 10.1186/s12882-022-02676-z

**Published:** 2022-01-25

**Authors:** Jia Neng Tan, Yi Da, Sabrina Haroon, Titus Lau

**Affiliations:** Division of Nephrology, University Medicine Cluster, National University Health System Republic of Singapore, Level 10, NUHS Tower Block, 1E Kent Ridge Road, Singapore, 119228 Republic of Singapore

**Correction to: BMC Nephrology (2022) 23:18**

**https://doi.org/10.1186/s12882-021-02646-x**

Following publication of the original article [1], the authors identified an error in the author name of Yi DA.

The incorrect author name is: Yi.

The correct author name is: Yi DA.

The author group has been updated above and the original article [1] has been corrected.
