# Supplementary material for: Chlorhexidine – a commonly used but often neglected culprit of dialysis associated anaphylactic reactions (case report)
Source: BMC Nephrol. 2022 Jan 6;23:18. doi: 10.1186/s12882-021-02646-x (PMC8734226; doi:10.1186/s12882-021-02646-x)
Supplement: Supplementary file 1 — Additional file 1. CARE Checklist of information to include when writing a case report. [file 12882_2021_2646_MOESM1_ESM.pdf]

CARE case report guidelines

CARE Checklist of information to include when writing a case report

(cc) BY-NC-ND

Topic	Item	Checklist item description	Reported on Line
Title	1	The diagnosis or intervention of primary focus followed by the words "case report"	Page 1, Line 2
Key Words	2	2 to 5 key words that identify diagnoses or interventions in this case report, including "case report"	Page 3, Line 4
Abstract (no references)	3a	Introduction: What is unique about this case and what does it add to the scientific literature?	Page 2, Line 8 - 23
	3b	Main symptoms and/or important clinical findings	Page 2, Line 8 – 23
	3c	The main diagnoses, therapeutic interventions, and outcomes	Page 2, Line 8 - 23
	3d	Conclusion—What is the main "take-away" lesson(s) from this case?	Page 3, Line 2 - 3
Introduction	4	One or two paragraphs summarizing why this case is unique (**may include references**)	Page 4, Line 12 - 20
Patient Information	5a	De-identified patient specific information.	Page 4, Line 22. Page 7, Line 16
	5b	Primary concerns and symptoms of the patient.	Page 4, Line 22. Page 7, Line 16
	5c	Medical, family, and psycho-social history including relevant genetic information	Page 4, Line 22. Page 7, Line 16
	5d	Relevant past interventions with outcomes	NA
Clinical Findings	6	Describe significant physical examination (PE) and important clinical findings.	Page 5, Line 6. Page 7, Line 21
Timeline	7	Historical and current information from this episode of care organized as a timeline	Table 1 and Table 2
Diagnostic Assessment	8a	Diagnostic testing (such as PE, laboratory testing, imaging, surveys).	Page 6, Line 14. Page 8, Line 5
	8b	Diagnostic challenges (such as access to testing, financial, or cultural)	NA
	8c	Diagnosis (including other diagnoses considered)	Page 7, Line 11. Page 8, Line 12
	8d	Prognosis (such as staging in oncology) where applicable	NA
Therapeutic Intervention	9a	Types of therapeutic intervention (such as pharmacologic, surgical, preventive, self-care)	Page 5, Line 12. Page 8, Line 9
	9b	Administration of therapeutic intervention (such as dosage, strength, duration)	Page 5, Line 12. Page 8, Line 9
	9c	Changes in therapeutic intervention (with rationale)	NA
Follow-up and Outcomes	10a	Clinician and patient-assessed outcomes (if available)	Page 7, Line 19. Page 10, Line 11
	10b	Important follow-up diagnostic and other test results	NA
	10c	Intervention adherence and tolerability (How was this assessed?)	NA
	10d	Adverse and unanticipated events	NA
Discussion	11a	A scientific discussion of the strengths AND limitations associated with this case report	Page 10, Line 12 – Page 12, Line 16
	11b	Discussion of the relevant medical literature **with references**.	Page 10, Line 12 – Page 12, Line 16
	11c	The scientific rationale for any conclusions (including assessment of possible causes)	Page 10, Line 12 – Page 12, Line 16
	11d	The primary "take-away" lessons of this case report (without references) in a one paragraph conclusion	Page 12, Line 6 - 10
Patient Perspective	12	The patient should share their perspective in one to two paragraphs on the treatment(s) they received	NA
Informed Consent	13	Did the patient give informed consent? Please provide if requested	Yes ☑ No ☐
